# How to Avoid a No-Deal ER Exit

**DOI:** 10.3390/cells8091051

**Published:** 2019-09-07

**Authors:** Tiziana Anelli, Paola Panina-Bordignon

**Affiliations:** 1 Vita-Salute San Raffaele University, 20132 Milan, Italy; 2 Division of Genetics and Cell Biology, IRCCS Ospedale San Raffaele, 20132 Milan, Italy; 3 Division of Neuroscience, IRCCS Ospedale San Raffaele, 20132 Milan, Italy

**Keywords:** endoplasmic reticulum, protein folding, ERGIC, traffic, COPII vesicles

## Abstract

Efficiency and fidelity of protein secretion are achieved thanks to the presence of different steps, located sequentially in time and space along the secretory compartment, controlling protein folding and maturation. After entering into the endoplasmic reticulum (ER), secretory proteins attain their native structure thanks to specific chaperones and enzymes. Only correctly folded molecules are allowed by quality control (QC) mechanisms to leave the ER and proceed to downstream compartments. Proteins that cannot fold properly are instead retained in the ER to be finally destined to proteasomal degradation. Exiting from the ER requires, in most cases, the use of coated vesicles, departing at the ER exit sites, which will fuse with the Golgi compartment, thus releasing their cargoes. Protein accumulation in the ER can be caused by a too stringent QC or by ineffective transport: these situations could be deleterious for the organism, due to the loss of the secreted protein, and to the cell itself, because of abnormal increase of protein concentration in the ER. In both cases, diseases can arise. In this review, we will describe the pathophysiology of protein folding and transport between the ER and the Golgi compartment.

## 1. Introduction

The mammalian endoplasmic reticulum (ER) is responsible for the folding and maturation of almost a third of the total cellular proteome, including almost all proteins destined for secretion or insertion into the plasma membrane. Besides, the ER houses the enzymes responsible for synthesizing the majority of steroids and lipids needed for cell-to-cell communications or for the biogenesis of membranes.

**Figure 1:**
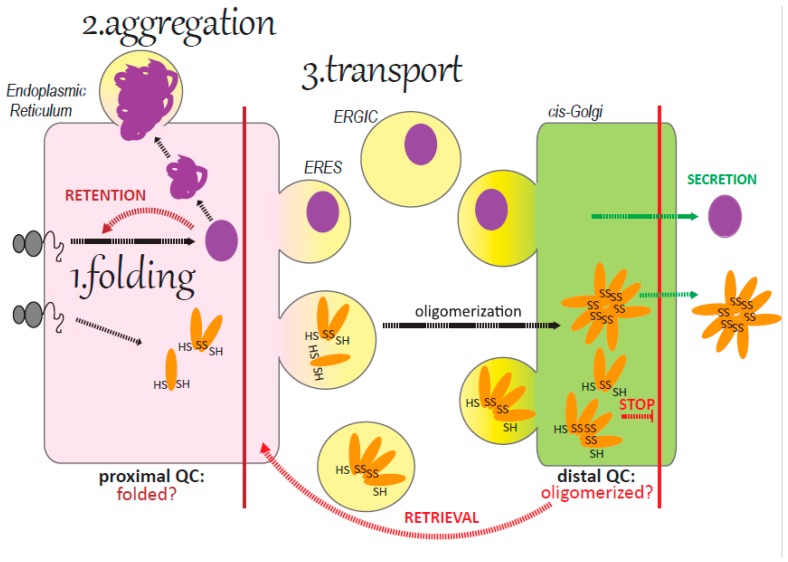
Protein folding, quality control (QC) and transport in the early secretory pathway. Only correctly folded proteins can pass the first step of QC located in the endoplasmic reticulum (ER) (proximal QC) and have access to transport vesicles at the ER exit sites (ERES). Unfolded proteins are instead retained and could eventually form aggregates. In the cisGolgi, a second step of QC (distal QC) ensures that only correctly assembled proteins can proceed along the secretory pathway, while assembly intermediates are retrieved to the ER for another chance of being incorporated into a polymer.

Secretory proteins are synthesized on ER bound ribosomes and attain their native conformation thanks to a plethora of specific ER folding factors and enzymes (chaperones, lectins, oxidoreductases) [1]. Only proteins that achieve their native structure pass the first step of protein quality control (QC) localized at the ER level (Figure 1). They are released by the ER folding factors, are inserted in COPII coated vesicles that bud from the ER exit sites (ERES), traverse the ER to Golgi intermediate compartment (ERGIC), and then proceed to the Golgi complex. In the ERGIC-cisGolgi compartment, a second step of QC is present, specifically dedicated to oligomeric proteins whose monomers are bound by disulfide bonds (adiponectin, IgM). A multifunctional soluble chaperone residing at this level (ERp44) recognizes and brings back to the ER assembly intermediates of soluble proteins, while only fully assembled proteins are released in the Golgi lumen [2,3]. As to membrane proteins, the transmembrane proteins RER1, localized at the cis-Golgi level, interacts with unassembled subunits of multimeric transmembrane proteins, retrieving them back to the ER [4,5]. 

### 1.1. Folding and Quality Control in the Early Secretory Pathway

Protein folding in the ER is extraordinarily challenging, as the ER must manage to modify polypeptides that can be present at very high concentration—up to 100 mg/mL—in the ER lumen [6]. Moreover, folding in the ER is slow and coupled to covalent disulfide formation, transmembrane insertion, N-glycosylation. Nonetheless, the ER displays an extraordinary capacity to assist folding and assembly of proteins at the different steps of protein maturation before they are sent out of the secretory pathway [7]. 

In animal cells, most secreted proteins are translocated into the ER co-translationally as unfolded nascent polypeptides. Thus, the rate of messenger RNA (mRNA) translation influences the burden of unfolded proteins in the ER. Quality control mechanisms exist that monitor synthesis of proteins as they are synthesized on the ribosomes. Alterations in the rate of translation, for example, allow for more accurate translation and folding [8]. The quality of the mRNA and its codon usage affect translation rates, and translation rate, in turn, alters chaperone binding to the nascent chain [9]. Perturbations in the ER can be sensed by the ER-resident transmembrane (PKR)-like ER kinase (PERK) that phosphorylates the eukaryotic initiation factor (eIF) 2α and transiently arrest translation, thus decreasing the flux of proteins entering the ER [7]. PERK activation is part of a specific stress response system, the unfolded protein response (UPR), that protects the ER folding environment by detecting and responding to the presence of misfolded proteins in its lumen [10]. 

The ER holds a vast repertoire of ER-resident chaperones and folding factors that direct and monitor each error-prone step in protein biosynthesis: post-translational modifications, oxidative folding, and maturation of client proteins to their functional tertiary or quaternary state [11,12]. In the ER, many secretory proteins undergo asparagine-linked glycosylation on specific sites (Asn/X/Ser-Thr). Once glycosylated, the two outer glucose units are co-translationally trimmed by glucosidases I and II generating a monoglycosylated asparagine-linked oligosaccharide, which is recognized by the lectin-like molecular chaperones calnexin and calreticulin. Release from these chaperones leads to enzymatic removal of the remaining glucose unit by ER glucosidase II. Conformational maturation releases glycoproteins from the protein-folding machinery, coupling this process to productive transport beyond the ER. 

Most accumulated and/or aggregated misfolded proteins are degraded by the endoplasmic reticulum-associated degradation (ERAD), while others escape the ER and are degraded instead by lysosomal proteases [13,14]. During ERAD, misfolded proteins are first recognized by ER and cytosolic chaperones and by chaperone-like lectins [11,15,16,17,18]. For glycosylated proteins, the processing of the N-glycan by the ER mannosidase I acts as a timer for protein fate decision [19]. Actually, the event of mannose trimming to degradation by ER mannosidase I likely occurs in specific sub-compartments of the ER, recently named quality control vesicles (QCVs) [20] or in the cisGolgi region [21], suggesting that some substrates of ERAD need to reach the Golgi before being degraded. Ubiquitinated proteins are then retro-translocated from the ER to the cytosol and delivered to the 26S proteasome for degradation [22,23,24].

### 1.2. Exiting the ER

**Figure 2:**
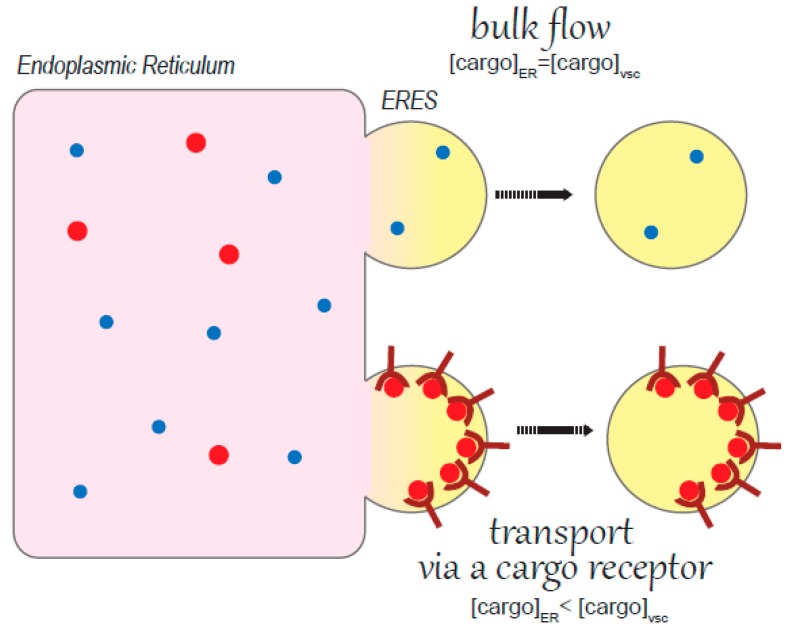
Mechanisms of exit from the ER. Proteins can be incorporated into vesicles departing from the ER simply by bulk flow or can be actively concentrated at the ERES by cargo receptors (active transport).

A hierarchy of quality control checkpoints sustains protein homeostasis in the ER so that only correctly folded proteins are released by the ER folding machineries and can proceed along the secretory pathway. Some proteins move forward following the so called bulk flow: the simple formation of vesicles exiting the ER creates a sucking force that, by chance, inserts proteins free to move into the vesicles departing from the ER [25] (Figure 2). In this case the concentration of proteins that are transported in the secretory vesicles is the same as that present in the ER lumen. Other proteins instead move thanks to active transport as proteins are recognized by specific cargo receptors: transporters, adaptors and components of the vesicle coat (Figure 2). These factors actively chose their binding cargo in the ER and insert them into the forming vesicles. In this case, the proteins to be transported are concentrated in the departing vesicles. Interestingly, all these mechanisms are such that the ER resident chaperones and enzymes are normally excluded from the departing vesicles. This is likely because ER folding factors exist as a supramolecular complex [26], important both for ensuring efficient folding on one hand and avoiding escape of enzymes by mistake on the other. Another hypothesis is that the action of cargo selection itself ensures exclusion of ER resident proteins from ER departing vesicles [27].

Vesicles exiting from the ER form at the so-called ER exit sites (ERES) [28,29]. Here, COPII components are recruited by protein adaptors to start the formation of the vesicles. The COPII coat is composed of different molecules, the small GTPase Sar1, the Sec23 and Sec24 subunits (which form heterodimers), and the Sec13/Sec31 subunits (which form heterotetramers) [30,31]. 

Transmembrane proteins that need to exit from the ER normally directly interact with the coat components. This interaction is mediated by specific amino acids in their cytosolic tail, which allows their specific recognition by the COPII components [32,33,34]. Some of them requires specific adaptors, as it is the case of AMPA receptors and TGFα. Their transport out of the ER is in fact mediated by the activity of cargo adaptors called Cornichon Proteins [35,36]. The length of the trans-membrane domain (TMD) itself is a constraint that allows the exit from the ER only of proteins with longer TMD, thus excluding from the departing vesicles transmembrane ER resident enzymes [37].

Few cargo receptors have been identified so far for secretory soluble proteins: ERGIC-53/LMAN1, and proteins with a similar structure such as VIPL, VIP36, ERGICL [reviewed in 28,38]. These are all transmembrane proteins that bind cargoes in the ER thanks to their luminal domain and interact with their cytosolic tail with COPII components. The interaction with the cargoes is generally mediated by glycan recognition, as all those transporters act as lectins [28,38]. 

Even if the majority of secreted proteins require the formation of COPII vesicles, non-conventional secretion cases have been described for selected cargoes, in which the exit from the ER is not dependent on COPII vesicles [39,40,41,42]. 

### 1.3. The COPII Cage

**Figure 3:**
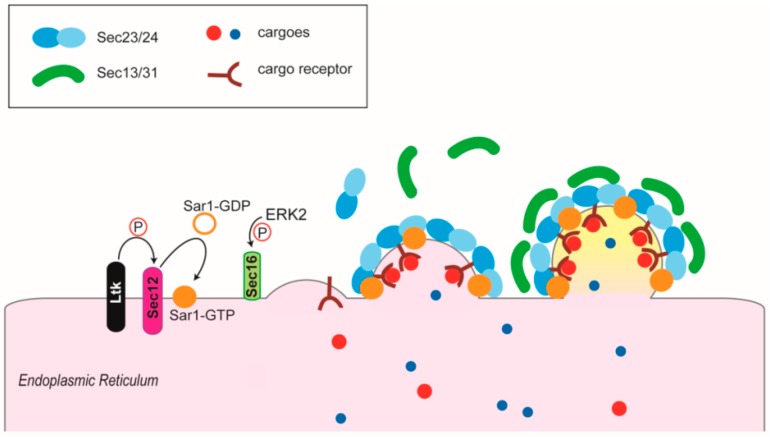
COPII assembly. The assembly of COPII vesicles is started by the activity of Sec12, which works as a nucleotide exchange factor for Sar1-GDP. Sar1-GTP is able to insert into the membrane of the ER at the ERES, recruiting first the Sec23/Sec24 heterodimer. The vesicle is completed with the addition of Sec13/Sec31. Cargo receptors or transmembrane cargoes can directly interact with Sec23/sec24 components. Sec12 can be activated by LTK. Sec16, another molecule part of the COPII coat, can be phosphorylated and hence activated by ERK2.

The COPII coat is a multimeric protein assembly whose formation is necessary for the secretion of the majority of proteins in eukaryotic cells (Figure 3) ([30,31] and references therein). Its assembly is initiated by the transmembrane protein Sec12, which activates the small GTPase Sar1. Sar1, in turn, inserts its N-terminal amphipathic helix in the ER membrane at the level of the ERES, initiating membrane curvature and recruiting the other COPII components, Sec23 and Sec24, through direct interaction with Sec23. Sec23 and Sec24 form a heterodimer that constitutes the inner part of the COPII coat and recognizes specific transport signals on transmembrane secretory proteins or protein adaptors. The assembly is completed with the recruitment of the second layer, composed by the Sec13 and Sec31 heterotetramer. The interaction occurs between Sec31 and Sar1 and Sec23 [29,43]. Another factor playing an important role in these processes is Sec16, an ER peripheral membrane protein that localizes at ERES independently from Sec23/24 or Sec13/31 [44]. Sec16 acts as a scaffold for COPII assembly [45,46]. 

An acute increase of cargo load in the secretory pathway often causes ER stress [7], under these conditions, mammalian cells show a decreased number of ERES, by reduced formation of COPII vesicles [47]. On the contrary, a chronic rise in cargo load induces increased size and numbers of ERES [48,49]. Moreover, the nature of the cargo can also regulate and modify the generation of COPII vesicles: some cargoes stimulate the Sar1GTPase activity of Sec23/Sec24, other can modify the structure of the Sec13/sec31 cage, influencing the geometry of the complex and hence the curvature of the vesicles that are budding from the ER membrane [31].

Interestingly, it has been recently described that the COPII machinery has another additional function: by selectively interacting with proteins that need to be transported, and protein adaptors, COPII actively sorts proteins that need to proceed along the secretory pathway. This is important to avoid the insertion in the departing vesicles of ER resident chaperones and enzymes, and of misfolded proteins [27].

How is the formation of vesicles departing from the ER controlled? The activity of Sec16 is required for ERES homeostasis [44] and it is controlled by MAPK signaling [50]. The Raf-MEK cascade, activated by growth factors in fact activates ERK2 which phosphorylates Sec16 stimulating COPII vesicles biogenesis [50]. In this way growth factors stimulation increases the levels of ER export [51]. Moreover, it has been very recently demonstrated that the activation of a tyrosine kinase present at the ERES (LTK) can phosphorylate Sec12, thus activating Sar1 and starting the assembly of the COPII coat [52] (Figure 3). How is LTK itself activated is still unknown, but this is for sure an interesting issue, as the correct, functional and efficient transport of secretory proteins out of the ER is a crucial step in protein homeostasis along the secretory pathway and, more in general, in the entire cell. 

### 1.4. What Happens in Case of No ER Exit?

The complexity of the protein maturation steps implies the existence of several machineries sequentially distributed along the secretory pathway, which must all work properly for proteins to reach their native state and their correct final localization. Should something go wrong in any of these steps, the protein will not be able, as a result, to be functionally active, likely leading to a pathological condition. 

Considering the protein maturation checkpoints in the early secretory pathway (ER, ERES, ERGIC), we can distinguish three different situations that could cause a disease linked to trafficking (Figure 1):

1. The QC (especially the proximal QC) is too stringent: this is the case for example of CFTR.
2. The protein folding is abnormal and the protein aggregates in the ER or the in ERGIC compartment: this is the case of some mutants of α1-anti trypsin (α1AT).
3. The protein trafficking between the ER and the Golgi is not efficient: this is the case of mutations in ERGIC-53, MCFD2, or mutations in components of the COPII coat.

## 2. Excessive Quality Control: The Case of Cystic Fibrosis Transmembrane Conductance Regulator (CFTR) 

Cystic fibrosis (CF) is the most common lethal monogenic autosomal recessive disease among the Caucasian population, and it is caused by dysfunction of the cystic fibrosis transmembrane conductance regulator (CFTR) glycoprotein, which normally functions as a chloride/bicarbonate channel at the apical membrane of epithelia [53,54,55]. Thus, its function is to maintain ion and fluid homeostasis. The human CFTR belongs to the ATP-binding cassette (ABC) transporter superfamily, and consists of 12-transmembrane domains (TMDs) that form the translocation pathway, two cytoplasmic nucleotide-binding domains (NBDs) that hydrolyze ATP, and a regulatory (R) domain that must be phosphorylated to allow the channel to open [55,56].

The most common CF-causing mutation is a deletion of phenylalanine 508 (ΔF508) present in approximately 85% of CF patients [57,58]. ΔF508 causes CFTR misfolding, which is recognized by the ER quality control system, resulting in ER retention and targeting for degradation by the ubiquitin-proteasome pathway [59]. The most severe consequences of the lack of CFTR activity manifest in the pancreas and in the lung. In the pancreas, the failure of bicarbonate-rich fluid and enzyme secretion impair intestinal digestion and absorption. In the lung, the defective Cl^−^ transport (thus fluid secretion) coupled with hyperabsorption of Na^+^ and fluid, leads to secretion of thick and dehydrated mucus and colonization by microorganisms causing damaging inflammatory responses. Multiple checkpoints at the level of folding and trafficking of the mutant ΔF508-CFTR have been suggested to regulate its ER retention and exit [59,60]. Folding checkpoints involve interaction with chaperones, and trafficking checkpoints involve recognition of trafficking/exit signals that direct towards vesicular transport [60,61,62]. 

Nascent ΔF508-CFTR is initially recognized by the Hsp70 machinery, which by interaction with the co-chaperone CHIP is converted from a protein folding system into a proteasomal degradation factor by mediating the covalent attachment of ubiquitin (E3 ubiquitin ligase activity) to chaperone substrate proteins [63]. A small fraction of ΔF508-CFTR that has escaped Hsp70 quality control is again assessed via recognition of its glycan moieties by a calnexin-independent mechanism. 

A trafficking checkpoint acts at the levels of recognition of the four three-residue arginine-framed tripeptides (AFTs) of CFTR, which function as an ER retention motif. Substitution of lysines for one of the arginines in each of these four triplets simultaneously allows nascent ΔF508-4RK-CFTR to mature about one-third and increase its function, although not fully to wild-type CFTR levels [64]. However, full correction of ΔF508-4RK-CFTR folding is not achieved as measured by the gating properties of the channel [65]. Release of ΔF508-CFTR from ER retention by interfering with recognition of AFTs signals may provide the basis of a novel therapeutic strategy for CF. VX-809 is a small molecule that repairs folding and processing defects of CFTR by promoting interactions between the first cytoplasmic loop (CL1) of transmembrane domain 1 (TMD1) and the first nucleotide-binding domain (NBD1), thus rescuing ΔF508-CFTR localization [62,66].

A further trafficking checkpoint occurs at the level of CFTR inclusion into COPII vesicles. Sorting of CFTR into vesicles budding from the ER is mediated by the interaction of the Sec23/24 subunits of COPII with a highly conserved diacidic code at residues 565–567 (sequence DAD) of CFTR [66]. Mutations in these residues severely affect export of CFTR. When the aspartate residue at 567 is replaced by an alanine (D567A), CFTR association with Sec24 is reduced [67] but folding is not affected [68]. Replacement of both aspartate residues with alanine (D565A, D567A; DD/AA-CFTR) blocks CFTR exit from the ER [62]. 

Progress has been made toward accomplishing the goal of effective CFTR modulator therapy. CFTR modulators include, among others, potentiators that promote channel activity, correctors (VX-809) that promote CFTR ER exit and traffic, and readthrough agents that restore full-length CFTR by suppression of premature termination codons. When combined with potentiators, correctors have been shown to sufficiently rescue CFTR in human bronchial epithelial cells (20–30% of CFTR activity) [69,70,71]. However, many patients with rare mutations are not yet impacted by CFTR modulators due to unknown susceptibility of their mutations to treatment. A growing knowledge of the molecular mechanisms responsible for defective CFTR suggests that an effective therapy should target repair of each phase of CFTR expression and function [72]. 

## 3. Aggregation of Misfolded Proteins in the ER or the in ERGIC Compartment: The Case of α-1 Anti-trypsin

α1-Antitrypsin (α1AT) is a member of the serine proteinase inhibitor (serpin) superfamily. The primary role of α1AT is to inhibit the proteolytic enzyme elastase secreted by activated neutrophils (NE), which degrade elastin within connective tissues. Thus, α1AT maintains the structural integrity of lung elastin [73,74]. 

α1AT is a single-domain protein of 394 amino acid residues, which fold into three β sheets and nine α helices that surround the β sheet scaffold [74]. A reactive center loop protruding from the main body of the molecule mediates α1AT inhibitory specificity [75]. Antitrypsin binds covalently to its target proteinase through specific residues within the RCL, and RCL translocates the proteinase to the opposite end. Due to this conformational change, α1AT inactivates the protease while α1AT is stabilized [76]. The metastable nature of α1AT is thus required to facilitate the rapid and gross conformational changes required for proteinase inhibition. Correct conformational maturation of the newly synthesized polypeptide is a prerequisite for its productive transport along compartments of the secretory pathway. During its biosynthesis in the ER, α1AT is subjected to a quality control checkpoint that initially facilitates the conformational maturation of newly synthesized α1AT [1]. 

Several naturally occurring genetic variants of α1AT have been identified, which associate with a diminished systemic concentration of the inhibitor causing α1AT Disease (α1ATD), an inherited disorder of the lung and liver [77,78]. A severely reduced concentration of α1AT ensues the elastolytic degradation of lung connective tissue, increasing the risk to develop chronic obstructive pulmonary disease (loss-of-function phenotype). Notably, as the main site for α1AT biosynthesis is the liver, the accumulation of mutated α1AT in the ER of hepatocytes is a risk factor for the development of childhood liver disease (gain-of-toxic function phenotype) [78]. 

The most frequent disease-associated mutations include a point mutation in the α1AT gene, which is named the ATZ variant [79,80]. The resulting single amino acid substitution Q342K leads to the slowdown of the last steps of folding [81], with consequent increased intracellular polymerization and accumulation of ATZ polymers in the ER of the hepatocytes. This causes ER stress, low secretion of α1AT, and eventually end-stage pathology requiring liver transplantation [73,82,83]. 

Intracellular polymers and insoluble aggregates of the mutant ATZ variant have been observed in the liver biopsy specimens from patients with ATD [74]. The cargo receptor ERGIC-53 described to be a cargo receptor for α1AT [84] (see paragraph 4.2.4), likely fails to recognize the misfolded, and likely polymerized, ATZ mutant, thus contributing to its further accumulation in the ER. The accumulation of ATZ in the ER is associates with markedly dilated ER cisternae, and very large vesicles [85]. 

Two main pathways are involved in the disposal of misfolded ATZ: the proteasomal pathway and autophagy. To avoid ER clogging ATZ is eliminated from the secretory pathway by the ER-associated degradation (ERAD) machinery that targets the misfolded protein into the cytosol for degradation by 26S proteasomes. Indeed, ATZ was one of the very first identified substrates of the ERAD pathway [86]. Autophagy was later described as an additional pathway for ATZ clearance [87]. Accumulation of ATZ within the ER (and ERGIC) is sufficient to activate the autophagic response as demonstrated by increased GFP^+^ autophagosomes in the liver of an autophagosome reporter mouse [88]. Notably, autophagy-enhancing drugs promote ATZ clearance and attenuate hepatic fibrosis [89,90]. Recently, an ER-to-lysosome degradation pathway has been described for proteasome resistant ATZ aggregates [91]. 

A peculiar characteristic of the cellular response to the ER accumulation of ATZ is the lack of significant activation of the unfolded protein response (UPR) [92]. Recent structural data provide an explanation for why soluble monomeric ATZ does not get recognized by the UPR apparatus: monomeric ATZ intermediate adopts a conformation that resembles the wild-type molecule and, therefore, would not be recognized as unfolded [93]. 

Gene expression profiling of transgenic mice with inducible expression of ATZ in the liver have shown marked up-regulation of the regulator of G protein signaling (RGS16) [94]. RGS16 did not increase upon exposure to classical inducers of ER stress, indicating that its activation is characteristic and specific of ATZ accumulation in the ER. Furthermore, targeted disruption of the heterotrimeric G protein Gαi3 in a mouse model leads to a marked increase in hepatic insulin-induced autophagic activity [95]. This data indicates that Gαi3 plays a role in the hepatic anti-autophagic effect of insulin. Since RGS16 binds to Gαi3 and likely inhibits G signaling, induction of RGS16 upon ATZ accumulation in the ER might inhibit a Gαi3-mediated signaling pathway and thus de-represses autophagy [96]. 

Currently, there is no cure for severe liver disease and the only management option is liver transplantation when liver failure is life-threatening. New therapies that target the misfolded a1AT or attempt to correct the underlying genetic mutation are currently under development. 

## 4. Defective Transport

### 4.1. COPII Cage and Diseases: The Cases of Craniolenticular Dysplasia (CLDS) and Dyserytropoietic Anemia Type II

Export of folded proteins from the ER is mediated, except a few cases, by COPII coated vesicles. Different genetic diseases originate from mutation (loss of sense or missense) in COPII components. Surprisingly, almost all these diseases are tissue-specific and cargo-specific. One should expect in fact that, given the crucial role for COPII in cargo export from the ER, mutations in one of the COPII components should be incompatible with life or at least should cause very severe diseases with effects spread all over the body. The first explanation for this selectivity is the existence of different paralogs of the COPII components in mammals: in mammalian cells, there are 2 Sar1 isoforms (Sar1 A and B), 2 Sec23 isoforms (Sec23 A and B), 4 Sec24 isoforms (Sec24 A, B, C, D), 2 Sec31 (Sec31 A and B), and 2 Sec16 (Sec16 A and B) isoforms. Moreover, not all of these variants are always present in all tissues and cells. Hence, the presence of different isoforms, with different tissue distributions (Human Protein Atlas) and different cargo specificity [43,97] can explain the specific restricted effects observed in the pathologies caused by mutants of COPII components and in knock-out (KO) animal models.

Mutations in Sec23A in humans are responsible for the autosomal recessive disease Craniolenticular dysplasia (CLDS), a rare syndrome manifesting with large and late closing fontanels and calvarial hypomineralization, skeletal defects, and hypertelorism and other facial dysmorphisms [98,99,100]. The disease is caused by a missense substitution (F382L) in Sec23A. Especially osteoblasts, which have very low levels of the Sec23 B isoform [100], suffer from the lack of activity of this protein. The result of the mutation is defective collagen export, with intracellular accumulation in the ER, which is hence characterized by dilated cisternae [99]. On the other hand, mutations in Sec23B is the cause of congenital dyserytropoietic anemia type II [101,102], as red blood cells have very low levels of the isoform Sec24A with respect to Sec23B and cannot face the lack of the isoform B. Very interestingly, KO mice for Sec23B die very soon after birth because of the degeneration of professional secretory tissues (pancreas, salivary glands, glands of the digestive tract). The different phenotypes observed between humans and mice can account for species-specific functions of Sec23B [103]. 

According to their crucial role in regulating protein export from the ER, COPII components need to be upregulated under those differentiation programs that physiologically transform a cell into a protein factory. This is the case of B to plasma cells differentiation. After the encounter with an antigen, resting B lymphocytes are activated and become protein factories devoted to immunoglobulin production and secretion. This transformation implies a complete rearrangement of their secretory pathway [104,105]. As expected, the ERES and COPII components are also upregulated during this process [106]. The increase of the factory capacity, in fact, has no effects (and could be even deleterious) if the flux of products exiting from the factory is not increased too. 

### 4.2. ERGIC-53 and MCFD2: The Case of F5F8D

#### 4.2.1. ERGIC-53

ERGIC-53 (also known as LMAN1, Lectin Mannose Binding Protein 1) is a transmembrane protein of about 53 kDa shuttling from the ER to the cis-Golgi [107] and as such considered as a marker of the ERGIC compartment. ERGIC-53 presents a luminal lectin domain, a transmembrane region, and a short cytosolic domain ending with the sequence KKFF. The double phenylalanine at the C-terminus is a specific sequence needed for interaction with Sec24 for ER export [108,109]. The double lysine instead are needed for interaction with COPI and retrieval from the ERGIC-cis Golgi back to the ER [110]. ERGIC-53 can form covalent dimers and hexamers via the formation of disulfide bonds at the levels of the stalk luminal domain. The formation of at least dimeric structures is needed for ERGIC-53 to interact with the COPII components [111] while the formation of the hexamers is not required for ERGIC-53 exiting from the ER [112]. The luminal domain recognizes high mannose N-glycans on proteins to be transported [113], thus ERGIC-53 receives correctly folded proteins exiting from the primary QC steps (specifically from the Calnexin-Calreticulin cycle). Binding to the substrates is pH and calcium-dependent. ERGIC-53 binds its substrates at almost neutral pH in the ER and releases them in the Golgi because of a lower pH (about 6,5). The pH sensitivity is ensured by a histidine residue in the core of the lectin-binding domain [114]. Thanks to this mechanism, it is thought that ERGIC-53 exits from the ER once it is cargo-loaded and comes back empty via COPI vesicles.

#### 4.2.2. F5F8 Deficiency

The first discovered substrates of ERGIC-53 were Factor V and Factor VIII of the blood coagulation cascade [115]. Mutations of ERGIC-53 are in fact correlated with 70% of the cases of Combined Deficiency of Factor 5 and Factor 8 (F5F8D), an autosomal recessive mild coagulation disorder characterized by very low levels in the blood of both Factor V and Factor VIII (between 5% and 20% of normality). The most common symptoms are epistaxis, menorrhagia, and excessive bleeding during or after trauma. Factor V and VIII are bulky proteins characterized by a huge number of N-glycosylations. The lack of ERGIC-53 slows down the velocity of exit from the ER (and hence secretion) of both Factor V and VIII [116]. The presence, however, of a low level of these proteins in the blood accounts for secretion via bulk flow, even if the action of other lectin transporters cannot be excluded.

#### 4.2.3. MCFD2

As discussed before, only 70% of F5F8D cases account for mutations in ERGIC-53. The remaining 30% is instead characterized by mutations in MCFD2 (Multiple Coagulation Factor Deficiency protein 2) [117]. MCFD2 is a small soluble protein of about 16kDa, which interacts with ERGIC-53. MCFD2 has two EF hand-sites, which are important for calcium-dependent interaction with ERGIC-53 and with cargoes [118,119]. Mutations in MCFD2 causing F5F8D are null mutations but also missense mutations, which cause destabilization of its flexible structure and the loss of its interaction with ERGIC-53 [120]. Both types of mutation result in the inability of ERGIC-53 to interact with FV and FVIII leading to disease onset, as MCFD2 is required for ERGIC-53 interaction with FV and FVIII. Using an immunological comparison, considering ERGIC-53 as an immunoglobulin heavy chain, we could state that MCFD2 behaves as its light chain.

#### 4.2.4. Other ERGIC-53 and MCFD2 Substrates

ERGIC-53 acts as a lectin transporter not only for FV and FVIII of the coagulation cascade but also for other proteins travelling along the secretory pathway. Both lysosomal enzymes cathepsin C and Z have been described as substrates of ERGIC-53 [108,121], as their localization in the lysosomes is reduced in the absence of the lectin or when mutants unable to exit the ER are expressed. Moreover, a physical interaction between ERGIC-53 and these lysosomal enzymes has been demonstrated [122].

Metallopreotease-9 (MMP9) has been found associated with ERGIC-53 (both in YFP complementation assays and after crosslinking) and its secretion is downregulated in ERGIC-53 KO cells [123].

In vitro and in vivo studies also indicated that α1AT is a substrate of the ERGIC-53/MCFD2 complex (see also paragraph 2). The intracellular trafficking of α1AT is delayed both in cells subjected to ERGIC-53 or MCFD2 KO [84]. ERGIC-53 and MCFD2 KO female mice show lower plasma α1AT levels and accumulation of α1AT in the hepatocytes, with enlarged ER [124,125]. Up to now, however, no α1AT patients have been isolated showing an ERGIC-53 mutation. It would be interesting to analyze whether the few α1AT patients with Z mutations, which develop hepatic disease, have mutations in ERGIC-53 or MCFD2.

We demonstrated in the past that a common substrate of the ERGIC-53/MCFD2 pair is the IgM Heavy Chain µ. ERGIC-53 and MCFD2 downregulation, in fact, decreases both IgM polymerization efficiency [126] and the intracellular accumulation of a mutant IgM heavy chain lacking the first constant domain [127]. We proposed that ERGIC-53, being a planar hexamer, could work as a platform for IgM polymerization during subunit transport from the ER to the Golgi compartment. As such, both ERGIC-53 and MCFD2 are increased during the differentiation of a B lymphocyte to a plasma cell. Interestingly, preliminary unpublished data indicate that F5F8D patients show lower levels of IgM compared to controls (Anelli, Sitia, Peyvandi unpublished data), although in the range of normality. This also suggests that in plasma cells, redundant mechanisms likely exist to ensure the correct assembly of such important and essential molecules as IgM.

The soluble secreted protein Mac-2BP, which promotes integrin-mediated cell adhesion, in also a substrate of the ERGIC-53/MCFD2 complex [128]. According to its role in binding to Mac-2BP and in ERGIC-53 role in MMP9 secretion, higher levels of MCFD2 were recently described in metastasis of human oral cancer [129]. This could be also a general characteristic of those cells that need to increase their secretory capacity.

Recently, it has been shown that ERGIC-53 also promotes the secretion of transmembrane proteins, the GABAaRs [130] and the GP glycoproteins of Arenavirus [131]: interestingly in these cases, the interaction seems to be glycan-independent.

## 5. Concluding Remarks

The components of the QC machinery in the early secretory pathway interact with a diverse array of cargoes and thus must find the best deal for allowing proteins to exit from the ER. Only folded proteins are given the permit to leave the ER and continue their journey along the secretory pathway. Misfolded proteins instead must be stopped and redirected to other destinations—the proteosome or the lysosome—not to accumulate in the ER and affect the organelle’s homeostasis. While this strategy protects the ER from proteotoxic stress, in some conditions an excessive QC could not allow a putative functional protein to reach its final destination, thus leading to a loss-of-function pathological condition. Further problems might arise when the protein is given the permit to leave the ER, but the transporters are not functional. Thus, on the one hand novel effective therapeutic approaches for diseases associated to defects of this route must be directed to multiple intracellular targets. On the other hand, increasing the current knowledge of the molecular mechanisms regulating protein traffic from the ER could have important perspectives for the cure of trafficking diseases.
